# StomachDB: An Integrated Multi-Omics Database for Gastric Diseases

**DOI:** 10.3390/biology14111484

**Published:** 2025-10-24

**Authors:** Gang Wang, Zhe Sun, Shiou Yih Lee, Mingyu Lai, Xiaojuan Wang, Sanqi An

**Affiliations:** 1 Research Center of Oncology, Guangxi Academy of Medical Sciences, Nanning 530021, China; gwang@gxams.org.cn; 2 Life Sciences Institute, Guangxi Medical University, Nanning 530021, China; 20224132636@sr.gxmu.edu.cn; 3 Guangxi Key Laboratory of AIDS Prevention and Treatment, School of Public Health, Guangxi Medical University, Nanning 530021, China; 4 Faculty of Health and Life Sciences, INTI International University, Nilai 71800, Malaysia; shiouyih.lee@newinti.edu.my; 5 Department of Gastroenterology, The First Affiliated Hospital of Guangxi Medical University, Shuangyong Road, Guangxi Zhuang Autonomous Region, Nanning 530021, China; laimingyu@sr.gxmu.edu.cn; 6 College of Life Sciences, Northwest University, Xi’an 710069, China; xiaojwang@nwu.edu.cn

**Keywords:** gastric diseases, cancer, multi-omics, database, therapeutic targets

## Abstract

**Simple Summary:**

Gastric diseases, including gastric cancer, gastritis and gastric ulcers, pose significant threats to global health. However, the study of their complex causes and the development of improved diagnostic and therapeutic strategies are hindered by a lack of integrated, multi-type biological data. Here, we have developed StomachDB—the first comprehensive database dedicated to gastric diseases. This database integrates 6 types of biological data, encompasses 44 gastric diseases in both humans and mice, and includes about 2.5 million integrated molecular profiles along with 268,000 links between diseases and genes. Additionally, it offers user-friendly tools for data exploration and the identification of potential treatment targets. StomachDB overcomes the limitations of single-type data studies, enhances understanding of gastric diseases, and accelerates the development of precise diagnosis and therapeutic approaches, ultimately benefiting public health.

**Abstract:**

Gastric diseases represent a significant challenge to global health. A comprehensive understanding of their complex molecular mechanisms, particularly the pathways of molecular progression in precancerous lesions, is essential for enhancing diagnosis and treatment. StomachDB, the first comprehensive multi-omics database dedicated to gastric diseases, has been developed to address these research needs. This database integrates 6 types of biological data: genomics, transcriptomics, emerging single-cell and spatial transcriptomics, proteomics, metabolomics, and therapeutic-related information. It encompasses 44 gastric-related pathologies, including various forms of gastric cancer, gastric ulcers, and gastritis, primarily involving humans and mice as model organisms. The database compiles approximately 2.5 million curated and standardized profiles, along with 268,394 disease-gene associations. The user-friendly analytics platform provides tools for browsing, querying, visualizing, and downloading data, facilitating systematic exploration of multi-omics features. This integrative approach addresses the limitations of single-omics analyses, such as data heterogeneity and insufficient analytical dimensions. Researchers can investigate the clinical significance of target genes (e.g., *CDH1*) across different omics levels and explore potential regulatory mechanisms. Furthermore, StomachDB emphasizes the discovery of therapeutic targets by cataloging interactions among chemical drugs, traditional herbal medicines, and probiotics. As an open-access resource, it serves as a powerful tool for studying complex biological interactions and regulatory mechanisms.

## 1. Introduction

Gastric health is recognized as critical for the normal functioning of the entire digestive system. A significant geographical heterogeneity in the global burden of gastric diseases has been observed, with increasing trends documented in specific regions [1]. Gastric cancer, as a major global health concern, is characterized by high incidence and mortality rates, particularly in East Asia [2]. Current research primarily focuses on elucidating the molecular mechanisms underlying gastric mucosal inflammation, precancerous lesions, and malignancies. *Helicobacter pylori* (*H. pylori*) infection has been identified as a key risk factor for gastric pathologies, affecting an estimated 50% of the global population [3]. Furthermore, chronic inflammatory conditions of the gastric mucosa, such as atrophic gastritis, have been shown to correlate closely with precancerous transformations [4]. Despite recent advances, several challenges persist. The precise alignment between molecular subtypes of gastric diseases and clinical phenotypes remains insufficiently established, particularly regarding the comprehensive delineation of molecular progression pathways in precancerous lesions [5]. Additionally, drug resistance mechanisms involve complex multilayered interactions, including epigenetic regulation and microbe–host interactions [6]. Future studies will require the integration of multi-omics data and interdisciplinary technologies to overcome limitations in spatial heterogeneity and temporal dynamics within gastric disease research [7,8,9,10]. This integrated approach is expected to uncover pathogenic mechanisms across multiple dimensions, thereby providing pivotal insights for precision diagnostics and effective therapeutics.

Multi-omics databases serve as critical research tools for unraveling the molecular mechanisms and regulatory patterns of complex diseases by integrating biological data from multiple dimensions, including genomics, transcriptomics, proteomics, and metabolomics, to construct comprehensive cross-omics global biological networks [11]. Compared to single-omics analyses, integrated strategies in multi-omics databases effectively address challenges such as data heterogeneity, limited sample sizes, and insufficient analytical dimensionality, thereby significantly enhancing research reliability and comprehensiveness [12]. For instance, the open cancer multi-omics database MLOmics has notably advanced the development and evaluation of bioinformatics and machine learning models [10]. MLOmics encompasses 8314 patient samples, across 32 cancer types, and includes 4 omics types, stratified features, and extensive benchmark data. By integrating multi-dimensional multi-omics technologies, MLOmics plays a pivotal role in three major areas: first, it facilitates early and precise diagnosis by identifying specific biomarkers, significantly enhancing the sensitivity and specificity of diagnoses; second, optimizing treatment strategies by providing scientific guidance for targeted therapy, immunotherapy, and combination therapy, thereby improving treatment efficacy; and third, enhancing prognostic assessment by accurately predicting patient survival outcomes and recurrence risks [13]. Similarly, a multi-omics graph knowledge representation method has demonstrated robust performance in predicting in-hospital outcomes for pneumonia patients, surpassing previous single-type omics models, classical machine learning methods, and traditional deep learning approaches [14]. Furthermore, the integration of multi-omics data through techniques such as Graph Convolutional Networks (GCN) and Multi-Layer Matrix Factorization (MLMF) has significantly improved disease classification accuracy, thereby providing valuable support for precision medicine research [11,12,15]. Additionally, cross-species knowledge bases such as IAnimal have markedly enhanced the accessibility and analytical efficiency of public data through standardized storage architectures and visualization tools [16]. In fundamental research, multi-omics databases play a pivotal role in reconstructing gene regulatory networks, resolving cellular heterogeneity, and discovering drug targets [17].

Building upon this research foundation, StomachDB has been developed as the first global high-quality, multidimensional multi-omics database specifically dedicated to gastric diseases. This resource integrates 6 biological data types, including genomics, transcriptomics, single-cell and spatial transcriptomics, proteomics, metabolomics, and therapeutic-related information, comprehensively covering 44 gastric pathologies across 2 model species (*Homo sapiens* and *Mus musculus*). A user-friendly analytical platform is provided, which systematically visualizes multi-omics features through interactive infographics and modular tools. Researchers can query and analyze the clinical significance of target genes across gastric diseases alongside multi-omics characteristics, enabling the preliminary determination of potential regulatory mechanisms. Furthermore, StomachDB emphasizes the discovery of therapeutic targets for chemical drugs and traditional medicine-based interventions against gastric pathologies. Outcomes of probiotic treatments are also incorporated to facilitate the exploration of disease intervention strategies. By uncovering the associations between gastric diseases and various biological molecules, such as genes, proteins, and metabolites, StomachDB serves as a powerful tool for elucidating disease mechanisms and holds significant translational potential for advancing precision medicine in gastroenterology.

## 2. Materials and Methods

### 2.1. Single-Cell and Spatial Transcriptome Data Collection and Reanalysis

Single-cell and spatial transcriptomic data were obtained from https://immucanscdb.vital-it.ch/ (Gene expression, accessed on 1 April 2024), http://www.bmibig.cn/scAPAdb/ (APA, accessed on 1 April 2024), https://gene.ai.tencent.com/SpatialOmics/ (Spatial Omics DataBase, accessed on 8 April 2024), and http://gepia2.cancer-pku.cn/#index (Gene Expression Profiling Interactive Analysis 2, accessed on 20 April 2024) [18,19,20,21]. Particular attention was paid to digestive system-related tissues, and a total of 12 publicly available single-cell transcriptomic datasets related to gastric or digestive system tissues were downloaded from https://immucanscdb.vital-it.ch/ (IMMUcan SingleCell RNAseq Database, accessed on 15 April 2024) [18]. The specific details of these datasets are demonstrated in the single-cell module of StomachDB. Low-quality genes were excluded through filtering procedures considering the number of identified genes, mitochondrial gene content, and total counts. Uniform Manifold Approximation and Projection (UMAP) plots were used to visualize the dimensionality-reduced data, and cell types in each cluster were identified based on known marker genes. Finally, the ggplot2 (v 2.0.0) package was used to visualize the expression levels of all gene features across different clusters. For spatial omics data, raw count matrices were first filtered to remove low-quality spots or barcodes based on criteria such as total UMI counts, gene detection rate, and mitochondrial gene content (>10% mitochondrial reads were excluded). The filtered data were then normalized by total counts, log-transformed, and scaled to mitigate library-size effects. When datasets contained more than 2000 features, the top 2000 highly variable genes were selected using the highly variable genes function (flavor = “seurat”). Dimensionality reduction was subsequently performed using PCA, followed by clustering through the FindClusters (5.2.1) function implementing the nearest neighbor clustering method. In combination with the GEPIA2 online database (accessed on 20 April 2024), gene features with |log2 (fold change)| > 1.5 were filtered.

### 2.2. Differential Expression Gene and Tissue-Specific Gene Analysis in Transcriptomics

Raw transcriptomic data were downloaded from the Gene Expression Omnibus (GEO, accessed on 3 August 2023), The Cancer Genome Atlas (TCGA, accessed on 3 August 2023), and Clinical Proteomic Tumor Analysis Consortium (CPTAC, accessed on 3 July 2023) databases for comprehensive gene expression and prognostic analysis [22,23]. Sequencing reads were mapped to the hg38 and mm10 genomes using HISAT2 (v 2.1.0) and StringTie (v 1.3.4), and transcript per million (TPM) values were calculated for genes, respectively [24,25]. Subsequently, differentially expressed genes (DEGs) analysis was performed using DESeq2 (v 1.42.0) [26,27]. The quantile normalization method in the R package “preprocessCore (v 1.64.0)” was applied to standardize gene expression levels across all samples, minimizing batch effects caused by different libraries. Genes with a false discovery rate (FDR) exceeding 0.05 were identified as DEGs [28]. Finally, the ggplot2 package (v 2.0.0) was used to visualize the acquired data.

### 2.3. Collection and Curation of Other Multi-Omics Data

The construction of StomachDB primarily integrates two strategies: public database mining and literature screening. Multiple internationally recognized biomedical databases were queried to obtain multi-dimensional data related to gastric diseases. The main data sources are as follows:

Genomics information associated with gastric diseases was retrieved from online databases including http://www.licpathway.net/ATACdb/ (Chromosome structure, accessed on 5 August 2023), https://ngdc.cncb.ac.cn/methbank/ (DNA methylation), https://www.encodeproject.org/ (Histone modification, accessed on 10 October 2023), https://www.cbioportal.org/ (Copy number alteration genes, mutated genes, structural variant genes, accessed on 12 November 2023), https://www.ncbi.nlm.nih.gov/snp/ (Single-nucleotide polymorphisms, SNPs, accessed on 28 November 2023), and https://www.omim.org/ (Online Mendelian Inheritance in Man, OMIM, accessed on 3 December 2023) [29,30,31,32,33]. Metabolomics data were downloaded from https://hmdb.ca/metabolites/ (accessed on 3 June 2024), where the search terms “gastric”, “stomach”, “stomach cancer” and “gastric cancer” were used to identify metabolites experimentally associated with gastric tissues or gastric diseases [34]. Proteomics data were obtained from https://www.uniprot.org/ (accessed on 10 March 2024), using the advanced search query ‘organism: Homo sapiens OR Mus musculus AND keyword: “gastric” OR “stomach” to collect protein entries linked to gastric tissues or gastric disease pathways. Each protein entry was extracted along with its gene name, protein name, function, and post-translational modification information, pubmed ID, etc. [35]. Treatment-related information for gastric diseases was collected from https://ctdbase.org/ (Chemical compounds, accessed on 15 March 2024) and http://www.tcmip.cn/ETCM/index.php/Home/Index/index.html (Chinese medicine, accessed on 3 September 2024) [36,37].

To capture associations between gastric diseases and probiotics, extensive searches were performed across literature databases in PubMed. Particular emphasis was placed on experimentally validated studies demonstrating links between probiotic interventions and specific gastric diseases or clinical phenotypes. The search strategy included the following terms: (“gastric disease” OR “gastritis” OR “gastric” OR “stomach” OR “stomach cancer” OR “gastric cancer”) AND (“probiotic” OR “*Lactobacillus*” OR “*Bifidobacterium*” OR “microbiome”). Transcriptomics-related information was sourced from http://www.splicedb.net/casa/ (Alternative splicing, accessed on 15 March 2024), https://www.ncbi.nlm.nih.gov/geo/ (Gene expression data), http://www.rnanut.net/lncrnadisease/index.php/ (Non-coding RNA, accessed on 4 October 2023), and https://rmvar.renlab.cn/#/home (RNA methylation, accessed on 25 October 2023) [38,39,40].

### 2.4. Data Inclusion/Exclusion Criteria, Batch Effect Correction, and Quality Control

All publicly available datasets incorporated in this study were sourced from high-quality, peer-reviewed repositories, including TCGA, CPTAC, GEO, HMDB and UniProt (the access date is as previously mentioned), to ensure the reliability and reproducibility of data sources. The inclusion criteria were as follows: (1) samples must be derived directly from human or murine gastric tissues, encompassing both tumor and non-tumor specimens; (2) data must be generated using standardized experimental platforms such as Illumina HiSeq 2000 (Illumina, San Diego, CA, USA), HiSeq X Ten (Illumina, San Diego, CA, USA), or LC-MS/MS (Agilent Technologies, Santa Clara, CA, USA); (3) raw data or preprocessed normalized expression matrices must be accessible, accompanied by complete metadata, including disease subtype, tissue source, experimental platform, and accession identifiers. Two categories of datasets were explicitly excluded: (1) datasets lacking essential metadata (e.g., tissue source or disease annotation); and (2) datasets containing fewer than six gastric tissue samples, to minimize potential statistical bias due to insufficient sample size.

Given the integration of multi-batch and multi-source data, particularly for transcriptomic and single-cell transcriptomic datasets, project-specific strategies were implemented to correct for batch effects. Considering the heterogeneity across disease types, experimental conditions, and sequencing batches, data from each project were analyzed independently to minimize inter-batch interference. For instance, transcriptomic datasets retrieved from GEO were processed individually. Each dataset was normalized to TPM format prior to differential expression analysis and visualization. For datasets involving multiple groups (e.g., disease stages or treatment interventions), pairwise comparisons (experimental group vs. control group) were performed to ensure the validity of intergroup analyses. For single-cell transcriptomic data, batch correction was conducted using the Harmony algorithm (v 1.1.0) when multiple samples originated from the same study, effectively removing technical batch variations. However, datasets from distinct gastric disease types were analyzed separately to avoid masking disease-specific molecular signatures. All datasets that met the inclusion criteria and passed batch effect correction were retained and integrated into the final StomachDB repository.

In terms of data quality control, as all data were obtained from internationally recognized biomedical databases that had undergone preliminary quality filtering, our quality control pipeline emphasized maintaining thematic relevance, metadata integrity, and comprehensive data coverage, rather than applying overly stringent statistical thresholds that might lead to data loss. The workflow proceeded as follows: First, during dataset retrieval from public repositories, a standardized set of search terms (“gastric disease” “gastritis” “gastric” “stomach” “stomach cancer” “gastric cancer”) was applied. Only datasets explicitly annotated as “gastric tissue-derived” or “gastric disease-related” in their titles, abstracts, or sample annotations were prioritized. Second, following initial screening, two independent researchers manually verified each dataset entry. The verification process assessed (1) the authenticity of tissue source, (2) the consistency between disease type and annotation, and (3) the completeness of key metadata. In cases of disagreement, a third senior researcher adjudicated the result by referring to the original publication and supplementary information from the database. Only datasets confirmed to be accurate were incorporated into StomachDB. Finally, only datasets with apparent outliers were subject to targeted exclusion; no additional restrictive thresholds were imposed, thereby ensuring maximal data retention and comprehensive analytical coverage.

### 2.5. Data Integration, Standardization, and ID Mapping

To ensure the consistency and compatibility of different omics datasets, several key steps were followed during the integration process: (1) Data Linking and Common Identifiers: Omics data were linked using gene symbols and Ensembl IDs as common identifiers. These identifiers facilitated the integration of genomic, transcriptomic, proteomic, and treatment-related multi-omics data, promoting their compatibility across different data types. (2) Data Normalization: To minimize technical biases and ensure comparability, various normalization methods were applied to different types of omics data. For transcriptomic data, raw RNA-seq data represented by counts and FPKM values were uniformly converted to TPM for normalization. For single-cell and spatial omics data, the raw data were standardized and then log-transformed, with a consistent threshold applied to filter out highly variable genes. For Chemical Compounds and Chinese Medicine data, gene names, inference networks, reference counts, and drug names were associated for easier comparison across diseases. For genomic data, values and scores provided by the source databases were used, as they were already standardized. (3) Integration Workflow: The standardized data were linked by common identifiers and structured into a relational database schema. The core of this schema consists of a set of fact tables (e.g., gene expression data) connected to dimension tables through foreign keys. This design enables efficient and complex querying across multiple omics levels, allowing users to seamlessly retrieve multi-omics features such as genomic, transcriptomic, and treatment-related data for specific genes.

### 2.6. Database Construction and Web Interface Implementation

Database Backend: Standardized and annotated data were used to construct a relational database, with MySQL 8.0 employed as the database management system. The database design adheres to the third normal form, comprising core data tables such as disease information, sample metadata, multi-omics data, and analysis results. Associations between data tables are established through primary keys (sample IDs, gene IDs) and foreign keys, enabling complex queries and data integration.

Web Server: The backend development utilizes the ThinkPHP 8.0.4 framework to build high-performance web services based on PHP (v 7.4.3). This framework provides RESTful API designs for implementing interfaces such as data query, analysis requests, and result export. The system employs SQLite (v 3.38.2) caching for frequently queried results to enhance response speed, integrates message queues to handle time-consuming analysis tasks, and maintains comprehensive records of system operation statuses and user activity logs. Backend services are deployed in a Apache2 (v 2.4.53) + PHP-FPM environment, with database access efficiency optimized via connection pooling technology.

Frontend Interface: The frontend interface is constructed using the Vue.js 3.0 framework to create interactive user experiences, following a three-layer design principle of “structure layer–presentation layer–behavior layer”. The code structure includes a public directory for configuration files and static resources, while the src directory contains subfolders such as assets for static resource files, components for global public components, and router for routing configurations. The core technology stack includes Vue.js 3.0 + Vue Router 4.2.4 as the view-layer framework, Element Plus (v 2.8.7) as the UI component library, Sass preprocessor + CSS Modules for styling, Axios for front-end to back-end data interaction, Pinia for global state management, and Vite 5.4.10 for fast compilation and hot reloading. The interface design adheres to modern web application standards, supporting responsive layouts compatible with both desktop and mobile devices.

## 3. Results

### 3.1. Construction Pipeline and Core Modules of StomachDB

**Figure 1:**
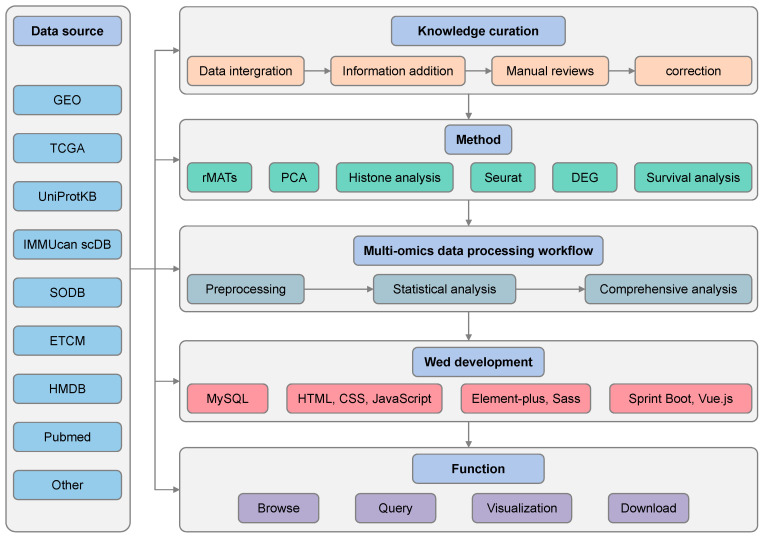
The workflow of data collection, processing, and database website construction for StomachDB.

The construction of StomachDB follows a standardized pipeline that begins with extensive data collection through both public database mining and manual literature curation. Subsequently, the collected heterogeneous data undergo rigorous preprocessing, standardization, and quality control to ensure consistency and accuracy. The curated and annotated data are then integrated into a MySQL-based relational database. A user-friendly web interface is ultimately developed to serve as the primary gateway for users to access and utilize the database resources. StomachDB has aggregated approximately 2.5 million integrated molecular profiles from 6 types of omics data, primarily focusing on human and mouse models, and covering 44 gastric-related diseases. The database integrates 243,094 genes from 38 tissues or cell lines and identifies various potential clinical treatments, including chemical components, traditional Chinese medicine, and probiotic formulations. To assist users in deciphering complex gene–disease–omics networks, StomachDB features four core functionalities: browsing, querying, visualization, and downloading (Figure 1).

### 3.2. Multifaceted Associations Between Gastric Diseases and Genes

Understanding the associations between genes and gastric diseases is critical for unraveling disease mechanisms and identifying potential therapeutic targets. StomachDB contains a total of 268,394 disease–gene associations that encompass major gastric-related diseases, including gastric cancer (such as gastric adenocarcinoma, diffuse gastric adenocarcinoma, gastroenteric adenocarcinoma, and microinvasive gastric cancer), gastric ulcer, and gastritis. Shared disease-related genes include *CDH1, COL4A1, TMPO, MYH11, ATRX,* among others. Notably, truncating or loss-of-function variants in the *CDH1* gene are the primary genetic drivers of hereditary diffuse gastric cancer, accounting for 25% to 50% of cases [41]. Inactivation of CDH1/E-cadherin occurs through epigenetic silencing or somatic mutations, which promote the invasiveness of diffuse gastric adenocarcinoma [42]. Additionally, MYH11 mutations are associated with dominantly inherited smooth muscle dysmotility syndromes, a cause of severe gastrointestinal motility disorders [43]. ATRX is responsible for maintaining the stability of DNA G4 structures by interacting with TERRA (telomere repeat-containing RNA); loss of ATRX function results in abnormal accumulation of DNA G4 structures, leading to replication stress and exacerbating chromosomal instability [44]. This genomic disorganization is recognized as a critical driver of the progression from gastric premalignant lesions, such as chronic atrophic gastritis, to gastric cancer [45]. These findings underscore the close relationship between gastric pathology and these genes.

**Figure 2:**
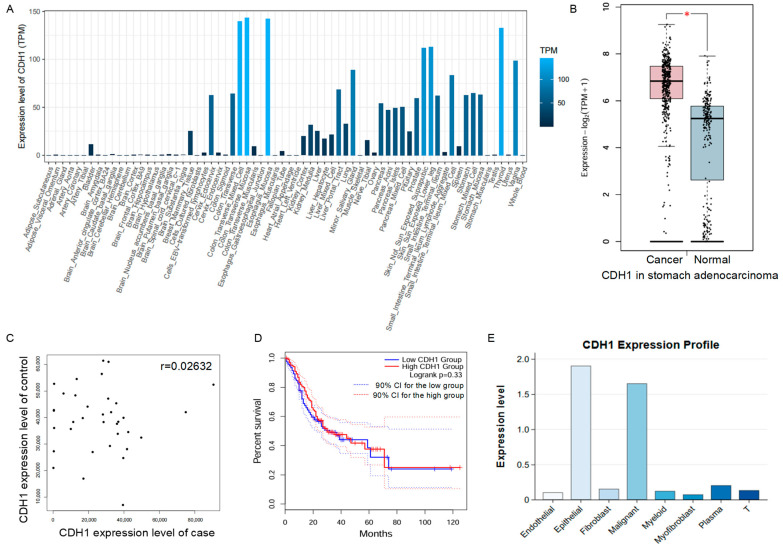
Main features and data modules of the StomachDB platform. (**A**) Expression levels of the *CDH1* gene across different human tissues, based on data from the GTEx project. (**B**) Differential expression of *CDH1* between gastric cancer tissues and adjacent normal tissues. * indicates statistical significance (*p* < 0.05). (**C**) Correlation analysis of CDH1 expression between gastric cancer and normal tissues. The *x*-axis represents *CDH1* expression levels in cancer tissues, and the *y*-axis in normal tissues. Original data were obtained from the GEO database. “r” refers to the correlation coefficient. (**D**) Survival analysis of CDH1. Patients were divided into high- and low-expression groups based on the median CDH1 expression level. The dashed line represents the 95% confidence interval (CI). (**E**) Expression landscape of *CDH1* across different cell types in gastric cancer tissues, derived from scRNA-seq data. The *x*-axis represents different cell clusters, and the *y*-axis shows the corresponding *CDH1* expression levels.

In StomachDB, users can efficiently retrieve detailed information using gene symbols from the homepage. For instance, by entering ‘CDH1’ and clicking the search icon, users are directed to comprehensive data about the gene, including its genomic location and functional characteristics, such as whether it acts as an RNA-binding protein or transcription factor (Supplementary Figure S1). This resource also provides insights into the expression patterns of *CDH1* across various tissues and omics layers (Figure 2A). Notably, *CDH1* shows a copy number deletion frequency of 1.90%, while its RNA expression level is significantly upregulated, indicating a weak correlation between copy number alterations (CNA) and RNA expression for this gene. (Figure 2B, Supplementary Table S1). Similar analyses can be conducted of other omics data. Furthermore, StomachDB offers information on gene sets with differential expression and prognostic relevance specifically for gastric adenocarcinoma patients (Figure 2C,D).

### 3.3. Classification of Gastric Diseases, Treatment Strategies, and Multi-Omics Data Across Species

To provide a comprehensive perspective for gastric disease research, StomachDB offers extensive coverage at the disease level, encompassing various types of gastric cancer, gastric ulcers, gastritis, and *H. pylori*-associated diseases. In terms of therapeutic information, StomachDB catalogs a total of 33,400 drug–disease interactions, which include 31,413 chemical compounds, 1950 traditional herbal medicines, and 37 probiotics (see Supplementary Tables S2–S4). Notably, cisplatin demonstrates high connectivity within the drug–disease–gene network, aligning with previous studies and thereby reinforcing the reliability and accessibility of StomachDB [46]. Regarding species coverage, the data are primarily derived from human and mouse samples, supplemented by additional information on pathogenic microorganisms, particularly probiotics. Importantly, StomachDB integrates a wide array of multi-omics data, which includes: (1) Genomics: Chromosome structure, DNA methylation, histone modifications, mutated genes, SNPs, structural variants, CNA, and OMIM virulence genes; (2) Transcriptomics: Alternative splicing, gene expression profiles, non-coding RNAs, and RNA methylation; (3) Proteomics; (4) Metabolomics; (5) Single-cell and spatial omics: gene expression, alternative polyadenylation (APA), and spatial histology. All results are presented in tabular format to facilitate intuitive and efficient queries. Users can retrieve relevant information by entering keywords or clicking on terms displayed at the top of the current page.

### 3.4. Spatial and Single-Cell Omics Data for Gastric Diseases

Given the critical role of cellular heterogeneity and the tissue microenvironment in the initiation and progression of gastric diseases, particularly gastric cancer, StomachDB incorporates emerging spatial transcriptomics and single-cell omics data. The gene expression homepage displays transcriptional signatures of gastric tissue genes across diverse cell types, including endothelial cells, fibroblasts, myeloid cells, and T cells (Figure 2E). Additionally, the database integrates select spatial transcriptomics data, providing insights into the spatial distribution of gene expression profiles within tissue sections. This integration facilitates mechanistic understanding of cell–cell interactions and the spatial architecture of pathological regions. The single-cell and spatial transcriptomics modules feature interactive plots for visualizing cell-type distributions and gene expression landscapes. For instance, users can select *CDH1* to visualize its spatial localization within gastric cancer tissue using UMAP and tissue heatmaps. When the *CDH1* gene is queried in the Spatial Histology section, its expression range (0–90) in mouse gastric cancer tissues is displayed, along with heatmaps of the corresponding annotation view and expression view for this gene. In the SC_UNB_10X_GSE134520 dataset under single-cell omics, information regarding the expression levels of the *CDH1* gene across different cell clusters can be retrieved through targeted searches. All data are organized into downloadable tables and linked to the original data sources, enabling convenient access and visualization for further analysis.

### 3.5. Interactive Visualization of Multi-Omics Profiles for Gastric Diseases

To facilitate the efficient integration and exploration of the multidimensional omics data stored in StomachDB, an intuitive and user-friendly homepage was designed to help users uncover hidden patterns and associations across various biological layers.

In the genomics module, StomachDB comprises three subcategories: Epigenome, Genomic Alteration, and OMIM Virulence Genes. The Epigenome section includes chromatin structure, DNA methylation (from both human and mouse samples), and histone modifications (H3K27me3, H3K27ac, H3K36me3, H3K4me1, H3K4me3, and H3K9me3; Supplementary Figure S2A). Under Genomic Alteration, StomachDB contains 107,069 CNAs, 71,173 mutation-associated gastric disease genes, 74,337 SNPs, and 2262 chromosomal structural variants, all of which are interactively visualized on the “Statistics” page (Supplementary Figure S2B,C).

In the metabolomics module, 11 key metabolites related to gastric cancer and atrophic gastritis are included, such as L-glutamic acid, pyruvate, cholesterol, and uric acid. The proteomics module provides detailed information on protein features, including functional domains, active sites, and experimentally annotated post-translational modifications (Supplementary Figure S2D,E). For example, when the *CDH1* gene is searched for in the Proteomics section, multidimensional information associated with this gene can be obtained. Specifically, this information includes: the name of the protein encoded by the gene, the name of diseases associated with this protein, the post-translational modification status, the biological function of the protein, the mechanism of protein activity regulation, the involved signaling pathways, and the PubMed IDs of relevant research studies.

For the transcriptomics module, 77 RNA-seq datasets (117 analyses in total) covering over 4.75 million transcriptomic profiles have been collected. These datasets span 10 gastric diseases, 3 species, and 32 body sites, with each transcriptomic profile subjected to both correlation and differential expression analysis. Moreover, transcriptomics data include non-coding RNAs, RNA methylation modifications, and alternative splicing events: Non-coding RNA data comprises 393 circRNAs, 745 lncRNAs, and 32 miRNAs (Supplementary Figure S2F); RNA methylation includes modifications such as 2′-O-Me, A-to-I, m1A, m5C, m5U, and m6A.

Collectively, the development of StomachDB enables researchers to systematically investigate the complex biological interactions and regulatory mechanisms underlying gastric diseases, thereby enhancing our understanding of gene regulation across multiple omics layers.

## 4. Discussion

Gastric diseases impose a substantial global health and economic burden. Deciphering their complex pathogenesis necessitates the integration of biological data across multiple hierarchical levels. In this context, we introduce StomachDB, the first comprehensive multi-omics database dedicated to gastric diseases, which is designed to provide an open-access resource for the global research community. StomachDB represents a significant integrative achievement in consolidating multi-dimensional data related to gastric diseases, with core advantages in focused targeting and cross-omics integration. Unlike general disease-oriented database platforms, StomachDB specifically focuses on the gastric ecosystem and its associated disorders, thereby minimizing data fragmentation and providing a specialized, precise, and comprehensive resource for gastric disease research. Notably, StomachDB is not only a static data repository but also a dynamic analytical platform that enables the generation and validation of cross-omics hypotheses. Its architecture supports the exploration of biological mechanisms across genomic, transcriptomic, and proteomic layers. For instance, users can examine the expression profiles of key gastric disease-related genes such as *CDH1*; integrate their genomic alterations, transcriptional dysregulation patterns, and proteomic post-translational modifications; and perform multi-dimensional association analyses to identify potential driver genes or regulatory axes. Additionally, by leveraging the built-in drug-related modules, users can further investigate potential therapeutic targets and explore drug repurposing opportunities by identifying key signaling nodes that mediate the link between genomic abnormalities and drug response pathways. Collectively, StomachDB serves as a cross-omics bridge that facilitates the exploration of molecular interactions underlying gastric diseases and supports target discovery and biomarker validation in translational research. The core value of StomachDB lies in its integration of diverse omics datasets, which encompass genomics, transcriptomics, microbiomics, and metabolomics, combined with treatment response data and robust literature-based evidence. Notably, the database includes emerging spatial transcriptomics and single-cell omics data, which enables researchers to investigate cellular heterogeneity and the tissue microenvironment of gastric diseases at high resolution. Furthermore, StomachDB incorporates curated literature on probiotic-based interventions for gastric disorders, thereby enriching its content and practical relevance. To enhance accessibility and usability, the database features a user-friendly interface and interactive visualization tools designed to lower barriers for researchers—including clinical practitioners specializing in gastric diseases, academic and biomedical scientists, educators, and students—as well as anyone interested in gastric research and its clinical applications, allowing them to effectively explore and utilize complex multi-omics data.

Existing multi-omics databases have provided essential data support and analytical foundations for biomedical research; however, they generally exhibit limitations when applied to gastric disease-specific studies. The construction of StomachDB was therefore aimed at addressing the insufficient applicability of current databases in this research domain. Specifically, while TCGA includes large-scale genomic and transcriptomic data across multiple cancer types, including gastric cancer, its scope is restricted to malignancies and lacks integration with proteomic and treatment-related datasets. In the field of gut-related research, databases such as GutUDB and gutMDisorder provide information on intestinal diseases and microbiome interactions, and resources like gutMDisorder, GMrepo, AMDB, and Twnbiome offer extensive microbial community data. However, these databases typically cover the entire digestive tract and lack specificity for the gastric microenvironment. Moreover, platforms such as GMrepo and AMDB exhibit limited integration of host multi-omics data, which constrains their utility in host–disease molecular network analyses [47,48,49,50]. Although GutUDB has taken a multi-omics approach to gut diseases, StomachDB offers deeper coverage and greater specificity for gastric disorders, making it a complementary resource in this space [51]. In contrast, StomachDB represents the first comprehensive multi-omics database specifically dedicated to gastric diseases and demonstrates several technical advantages. First, it achieves multi-dimensional data integration by consolidating six omics layers—genomics, transcriptomics, proteomics, metabolomics, single-cell/spatial transcriptomics, and drug-related data—into a unified analytical platform. Second, it expands data coverage by simultaneously incorporating gastric disease-related datasets from both humans and mice. Third, it enhances analytical practicality by providing interactive visualization tools and precise query modules. Moreover, StomachDB emphasizes translational applicability by incorporating therapeutic regimen and probiotic association information, effectively bridging molecular mechanism research with clinical translation. Collectively, compared with existing biomedical databases, StomachDB exhibits a unique positioning in the field of gastric disease research. Its disease-specific focus and integrative multi-omics architecture establish a systematic and specialized platform that supports in-depth exploration of gastric disease biology and the discovery of potential therapeutic targets. A direct comparison of features between StomachDB and other relevant databases is provided in Supplementary Table S5.

Despite its strengths, StomachDB currently faces several limitations. First, the database content largely depends on the availability of public datasets, which may result in uneven data coverage across disease types, sample sources, or omics layers. Second, the integration of heterogeneous data from different studies that utilize varying technical platforms and processing pipelines presents significant challenges. In this study, bulk RNA-seq data were employed for transcriptomic profiling due to their compatibility with normalization and cross-study comparison. Nevertheless, batch effects and platform-related biases may still persist. Third, while manual curation of literature and omics data enhances data reliability and interpretability, it is a time-consuming and labor-intensive process, potentially limiting the database’s ability to keep pace with the rapid growth in scientific publications and high-throughput technologies. Fourth, as a newly developed resource, the long-term maintenance and continuous updates of StomachDB will necessitate substantial and sustained investment of resources. Another limitation of the first version of StomachDB is that the metabolomics section on the website currently only includes gastric disease-related metabolites, without linking to specific genes—this is due to the lack of high-quality gene–gastric metabolite association data available for integration. We will address this in future updates by collecting and validating such association data, and optimize the website module to support gene–metabolite association queries.

To address these challenges and enhance the utility of StomachDB, several future development directions have been planned. We intend to update the database annually by incorporating newly released public datasets and studies related to gastric diseases. The update process will primarily rely on the development team for data curation and quality control. Each update will be released as a new version (e.g., StomachDB v1.1), accompanied by a changelog summarizing newly added datasets, features, and bug fixes. The homepage of the database will display the current version number and release date to ensure transparency. Moreover, we aim to expand the range of data types, with a particular focus on integrating more comprehensive single-cell and spatial transcriptomics data, detailed drug response profiles, and gastric imaging features. We also envision employing natural language processing and machine learning techniques to facilitate literature mining and knowledge extraction, thereby enhancing both curation efficiency and data comprehensiveness. Concurrently, we will continue to optimize interactive visualization tools, especially those designed for spatial and single-cell data, to ensure a more intuitive and user-friendly experience for exploration and analysis. In summary, StomachDB serves as a valuable foundation for advancing research into gastric diseases. Through continuous content expansion and functional enhancement, we are dedicated to developing StomachDB into a more comprehensive, powerful, and indispensable platform that facilitates deeper insights into the pathogenesis of gastric disorders and ultimately accelerates the discovery of novel diagnostic and therapeutic strategies.

## 5. Conclusions

As the first comprehensive multi-omics database dedicated to gastric disease research, StomachDB addresses the gap in the integration of specialized resources in this field by consolidating 6 major categories of data: genomics, transcriptomics, single-cell/spatial transcriptomics, proteomics, metabolomics, and therapeutic-related information. The database encompasses 44 types of gastric pathologies in both humans and mice, integrating approximately 2.5 million molecular profiles and 268,394 disease–gene associations. Its user-friendly analytical tools enable researchers to overcome the limitations of single-omics analysis, delve deeper into disease mechanisms, and identify therapeutic targets related to chemical drugs, traditional Chinese medicine, and probiotics, thereby making it a significant complement to existing gastrointestinal disease databases. StomachDB will continue to be optimized and upgraded by expanding data types and refining analytical tools, which is anticipated to enhance the understanding of the pathogenesis of gastric diseases and accelerate the translation of basic research into precise diagnoses and clinically effective treatments.

## Data Availability

The data are obtained through the public database. If a reasonable request is made, the corresponding author will provide information about the techniques used in this research. The URL of StomachDB is: http://stomach.henbio.cn/#/home (accessed on 23 October 2025).

## Supplementary Materials

The following supporting information can be downloaded at: https://www.mdpi.com/article/10.3390/biology14111484/s1, Supplementary Figure S1. Detailed gene information displayed in StomachDB following a search query. The top panel shows basic annotations returned after querying *CDH1*, including RBPs, therapeutic targets, and motif information. The bottom panel displays the tissue-specificity score of *CDH1*, based on data from the GTEx project. Supplementary Figure S2. Interactive visualization of multi-omics profiles associated with gastric diseases. (A) Pie chart summarizing six types of histone modifications. (B) Pie chart showing the distribution of genomic alterations. (C) Top 10 genes ranked by SNP count. The *x*-axis indicates the number of SNPs per gene, while the *y*-axis lists the top 10 genes; APC exhibits a markedly higher SNP count than the others, followed by genes in descending order. (D) Pie chart of 11 key metabolites associated with gastric cancer and atrophic gastritis. (E) Pie chart illustrating the functional features of proteins related to gastric diseases. (F) Pie chart and bar plots representing non-coding RNA profiles in the transcriptomic data, including the top 10 most abundant miRNAs, circRNAs, and lncRNAs. Supplementary Table S1. Copy number alterations of *CDH1* in different gastric-related diseases. Supplementary Table S2. The chemical compound information included in the StomachDB database. Supplementary Table S3. The Chinese medicine information included in the StomachDB database. Supplementary Table S4. The probiotic information contained in the StomachDB database. Supplementary Table S5. Comparison of features across StomachDB and representative existing databases.

## References

[1] Wang Y., Huang Y., Chase R.C., Li T., Ramai D., Li S., Huang X., Antwi S.O., Keaveny A.P., Pang M. (2023). Global Burden of Digestive Diseases: A Systematic Analysis of the Global Burden of Diseases Study, 1990 to 2019. Gastroenterology.

[2] Yuan W., Shi Y., Dai S., Deng M., Zhu K., Xu Y., Chen Z., Xu Z., Zhang T., Liang S. (2024). The role of MAPK pathway in gastric cancer: Unveiling molecular crosstalk and therapeutic prospects. J. Transl. Med..

[3] Duan Y., Xu Y., Dou Y., Xu D. (2025). Helicobacter pylori and gastric cancer: Mechanisms and new perspectives. J. Hematol. Oncol..

[4] Ben-Baruch Morgenstern N., Shoda T., Rochman Y., Caldwell J.M., Collins M.H., Mukkada V., Putnam P.E., Bolton S.M., Felton J.M., Rochman M. (2023). Local type 2 immunity in eosinophilic gastritis. J. Allergy Clin. Immunol..

[5] Tong Q.Y., Pang M.J., Hu X.H., Huang X.Z., Sun J.X., Wang X.Y., Burclaff J., Mills J.C., Wang Z.N., Miao Z.F. (2024). Gastric intestinal metaplasia: Progress and remaining challenges. J. Gastroenterol..

[6] Dong L., Zhu J., Deng A., Wei J., Li J., Mao X., Jia Z. (2023). Relationship between histone demethylase LSD family and development and prognosis of gastric cancer. Front. Immunol..

[7] Ni T., Wang H., Zhan D., Tao L., Lv M., Wang W., Chu Z., Zhou Z., Sunagawa M., Liu Y. (2021). CD133+/CD166+ human gastric adenocarcinoma cells present the properties of neoplastic stem cells and emerge more malignant features. Life Sci..

[8] Wang J.B., Gao Y.X., Ye Y.H., Zheng Q.L., Luo H.Y., Wang S.H., Zhang T., Jin Q.W., Zheng C.H., Li P. (2024). Comprehensive multi-omics analysis of pyroptosis for optimizing neoadjuvant immunotherapy in patients with gastric cancer. Theranostics.

[9] Sun C., Wang A., Zhou Y., Chen P., Wang X., Huang J., Gao J., Wang X., Shu L., Lu J. (2023). Spatially resolved multi-omics highlights cell-specific metabolic remodeling and interactions in gastric cancer. Nat. Commun..

[10] Yang Z., Kotoge R., Piao X., Chen Z., Zhu L., Gao P., Matsubara Y., Sakurai Y., Sun J. (2025). MLOmics: Cancer Multi-Omics Database for Machine Learning. Sci. Data..

[11] Li M., Chen Z., Deng S., Wang L., Yu X. (2024). MOSDNET: A multi-omics classification framework using simplified multi-view deep discriminant representation learning and dynamic edge GCN with multi-task learning. Comput Biol. Med..

[12] Yi H., Yang Q., Repaci C., Lee C.M., Heo G., Timsina J., Gorijala P., Yang C., Budde J., Wang L. (2024). TOPMed imputed genomics enhances genomic atlas of the human proteome in brain, cerebrospinal fluid, and plasma. Sci. Data..

[13] Zhou K., Yang C., Li Y. (2025). Multi-omics in colorectal cancer liver metastasis: Applications and research advances. Cancer Biol. Med..

[14] Zhou K., Yang C., Li Y. (2025). Multi-Omics Graph Knowledge Representation for Pneumonia Prognostic Prediction. IEEE J. Biomed. Health Inform..

[15] Ren Y., Ren F., Yang B. (2025). Multi-layer matrix factorization for cancer subtyping using full and partial multi-omics dataset. Brief. Bioinform..

[16] Tong L., Shi W., Isgut M., Zhong Y., Lais P., Gloster L., Sun J., Swain A., Giuste F., Wang M.D. (2024). Integrating Multi-Omics Data With EHR for Precision Medicine Using Advanced Artificial Intelligence. IEEE Rev. Biomed. Eng..

[17] Zhang H.W., Lv C., Zhang L.J., Guo X., Shen Y.W., Nagle D.G., Zhou Y.D., Liu S.H., Zhang W.D., Luan X. (2021). Application of omics- and multi-omics-based techniques for natural product target discovery. Biomed. Pharmacother..

[18] Camps J., Noël F., Liechti R., Massenet-Regad L., Rigade S., Götz L., Hoffmann C., Amblard E., Saichi M., Ibrahim M.M. (2023). Meta-Analysis of Human Cancer Single-Cell RNA-Seq Datasets Using the IMMUcan Database. Cancer Res..

[19] Zhu S., Lian Q., Ye W., Qin W., Wu Z., Ji G., Wu X. (2022). scAPAdb: A comprehensive database of alternative polyadenylation at single-cell resolution. Nucleic Acids Res..

[20] Yuan Z., Pan W., Zhao X., Zhao F., Xu Z., Li X., Zhao Y., Zhang M.Q., Yao J. (2023). SODB facilitates comprehensive exploration of spatial omics data. Nat. Methods.

[21] Tang Z., Kang B., Li C., Chen T., Zhang Z. (2019). GEPIA2: An enhanced web server for large-scale expression profiling and interactive analysis. Nucleic Acids Res..

[22] Wang Z., Jensen M.A., Zenklusen J.C. (2016). A Practical Guide to The Cancer Genome Atlas (TCGA). Methods Mol. Biol..

[23] Whiteaker J.R., Halusa G.N., Hoofnagle A.N., Sharma V., MacLean B., Yan P., Wrobel J.A., Kennedy J., Mani D.R., Zimmerman L.J. (2014). CPTAC Assay Portal: A repository of targeted proteomic assays. Nat. Methods.

[24] Kim D., Paggi J.M., Park C., Bennett C., Salzberg S.L. (2019). Graph-based genome alignment and genotyping with HISAT2 and HISAT-genotype. Nat. Biotechnol..

[25] Pertea M., Pertea G.M., Antonescu C.M., Chang T.C., Mendell J.T., Salzberg S.L. (2015). StringTie enables improved reconstruction of a transcriptome from RNA-seq reads. Nat. Biotechnol..

[26] Anders S., Pyl P.T., Huber W. (2015). HTSeq—A Python framework to work with high-throughput sequencing data. Bioinformatics.

[27] Love M.I., Huber W., Anders S. (2014). Moderated estimation of fold change and dispersion for RNA-seq data with DESeq2. Genome Biol..

[28] Bolstad B.M. (2024). PreprocessCore: A Collection of Pre-Processing Functions. https://github.com/bmbolstad/preprocessCore.

[29] Wang F., Bai X., Wang Y., Jiang Y., Ai B., Zhang Y., Liu Y., Xu M., Wang Q., Han X. (2021). ATACdb: A comprehensive human chromatin accessibility database. Nucleic Acids Res..

[30] Zhang M., Zong W., Zou D., Wang G., Zhao W., Yang F., Wu S., Zhang X., Guo X., Ma Y. (2023). MethBank 4.0: An updated database of DNA methylation across a variety of species. Nucleic Acids Res..

[31] ENCODE Project Consortium (2012). An integrated encyclopedia of DNA elements in the human genome. Nature.

[32] Gao J., Aksoy B.A., Dogrusoz U., Dresdner G., Gross B., Sumer S.O., Sun Y., Jacobsen A., Sinha R., Larsson E. (2013). Integrative analysis of complex cancer genomics and clinical profiles using the cBioPortal. Sci. Signal..

[33] Amberger J.S., Hamosh A. (2017). Searching Online Mendelian Inheritance in Man (OMIM): A Knowledgebase of Human Genes and Genetic Phenotypes. Curr. Protoc Bioinform..

[34] Wishart D.S., Guo A., Oler E., Wang F., Anjum A., Peters H., Dizon R., Sayeeda Z., Tian S., Lee B.L. (2022). HMDB 5.0: The Human Metabolome Database for 2022. Nucleic Acids Res..

[35] UniProt Consortium (2023). UniProt: The Universal Protein Knowledgebase in 2023. Nucleic Acids Res..

[36] Davis A.P., Wiegers T.C., Johnson R.J., Sciaky D., Wiegers J., Mattingly C.J. (2023). Comparative Toxicogenomics Database (CTD): Update 2023. Nucleic Acids Res..

[37] Xu H.Y., Zhang Y.Q., Liu Z.M., Chen T., Lv C.Y., Tang S.H., Zhang X.B., Zhang W., Li Z.Y., Zhou R.R. (2019). ETCM: An encyclopaedia of traditional Chinese medicine. Nucleic Acids Res..

[38] Burset M., Seledtsov I.A., Solovyev V.V. (2001). SpliceDB: Database of canonical and non-canonical mammalian splice sites. Nucleic Acids Res..

[39] Lin X., Lu Y., Zhang C., Cui Q., Tang Y.D., Ji X., Cui C. (2024). LncRNADisease v3.0: An updated database of long non-coding RNA-associated diseases. Nucleic Acids Res..

[40] Luo X., Li H., Liang J., Zhao Q., Xie Y., Ren J., Zuo Z. (2021). RMVar: An updated database of functional variants involved in RNA modifications. Nucleic Acids Res..

[41] Luo X., Li H., Liang J., Zhao Q., Xie Y., Ren J., Zuo Z. (2023). Genotype-first approach to identify associations between CDH1 germline variants and cancer phenotypes: A multicentre study by the European Reference Network on Genetic Tumour Risk Syndromes. Lancet Oncol..

[42] Zou G., Huang Y., Zhang S., Ko K.P., Kim B., Zhang J., Venkatesan V., Pizzi M.P., Fan Y., Jun S. (2024). E-cadherin loss drives diffuse-type gastric tumorigenesis via EZH2-mediated reprogramming. J. Exp. Med..

[43] Gilbert M.A., Schultz-Rogers L., Rajagopalan R., Grochowski C.M., Wilkins B.J., Biswas S., Conlin L.K., Fiorino K.N., Dhamija R., Pack M.A. (2020). Protein-elongating mutations in MYH11 are implicated in a dominantly inherited smooth muscle dysmotility syndrome with severe esophageal, gastric, and intestinal disease. Hum. Mutat.

[44] Tsai R.X., Fang K.C., Yang P.C., Hsieh Y.H., Chiang I.T., Chen Y., Lee H.G., Lee J.T., Chu H.C. (2022). TERRA regulates DNA G-quadruplex formation and ATRX recruitment to chromatin. Nucleic Acids Res..

[45] Xu W., Jiang T., Shen K., Zhao D., Zhang M., Zhu W., Liu Y., Xu C. (2023). GADD45B regulates the carcinogenesis process of chronic atrophic gastritis and the metabolic pathways of gastric cancer. Front. Endocrinol..

[46] Dasari S., Tchounwou P.B. (2014). Cisplatin in cancer therapy: Molecular mechanisms of action. Eur. J. Pharmacol..

[47] Dai D., Zhu J., Sun C., Li M., Liu J., Wu S., Ning K., He L.J., Zhao X.M., Chen W.H. (2022). GMrepo v2: A curated human gut microbiome database with special focus on disease markers and cross-dataset comparison. Nucleic Acids Res..

[48] Qi C., Cai Y., Qian K., Li X., Ren J., Wang P., Fu T., Zhao T., Cheng L., Shi L. (2023). gutMDisorder v2.0: A comprehensive database for dysbiosis of gut microbiota in phenotypes and interventions. Nucleic Acids Res..

[49] Yang J., Park J., Jung Y., Chun J. (2022). AMDB: A database of animal gut microbial communities with manually curated metadata. Nucleic Acids Res..

[50] Chattopadhyay A., Lee C.Y., Lee Y.C., Liu C.L., Chen H.K., Li Y.H., Lai L.C., Tsai M.H., Ni Y.H., Chiu H.M. (2023). Twnbiome: A public database of the healthy Taiwanese gut microbiome. BMC Bioinform..

[51] Bao Y., Chen Y., Lin L., Li J., Liu X., Wang G., Li Y., Lin Y., Chen Y., Zhou L. (2024). GutUDB: A comprehensive multiomics database for intestinal diseases. Imeta.

