# Supplementary material for: StomachDB: An Integrated Multi-Omics Database for Gastric Diseases
Source: Biology (Basel). 2025 Oct 24;14(11):1484. doi: 10.3390/biology14111484 (PMC12650210; doi:10.3390/biology14111484)
Supplement: Supplementary file 1 [file biology-14-01484-s001.zip › biology-3898419-supplementary.pdf]

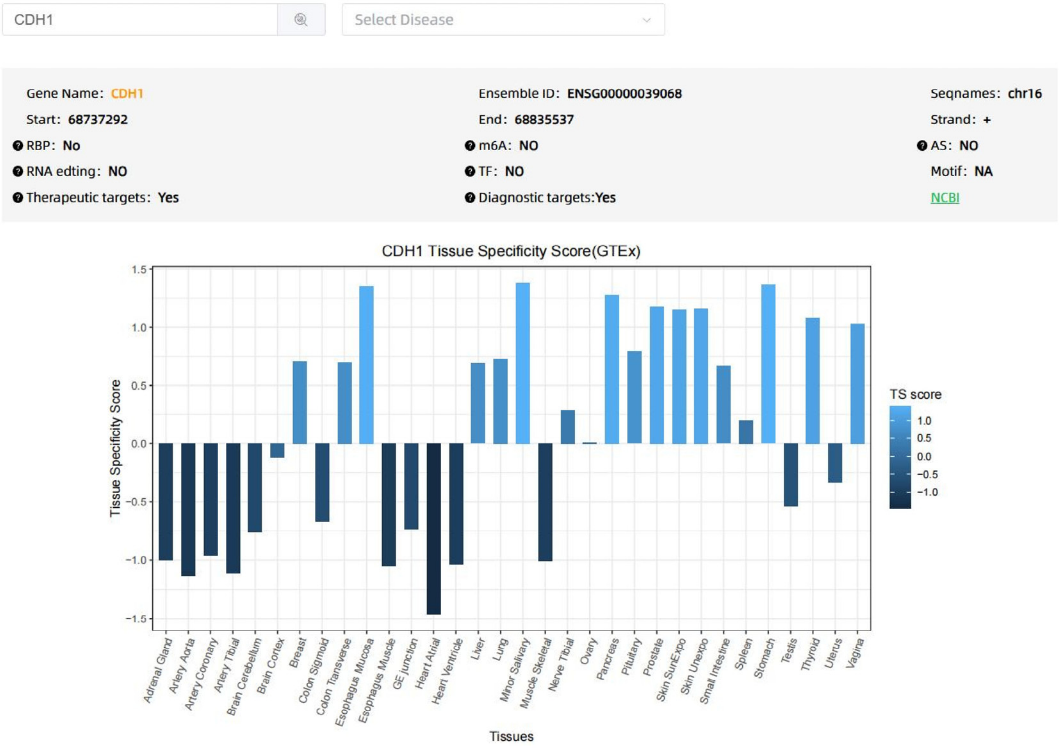

Supplementary Figure S1. Detailed gene information displayed in StomachDB following a search query. The top panel shows basic annotations returned after querying CDH1, including RBPs, therapeutic targets, and motif information. The bottom panel displays the tissue-specificity score of CDH1, based on data from the GTEx project.

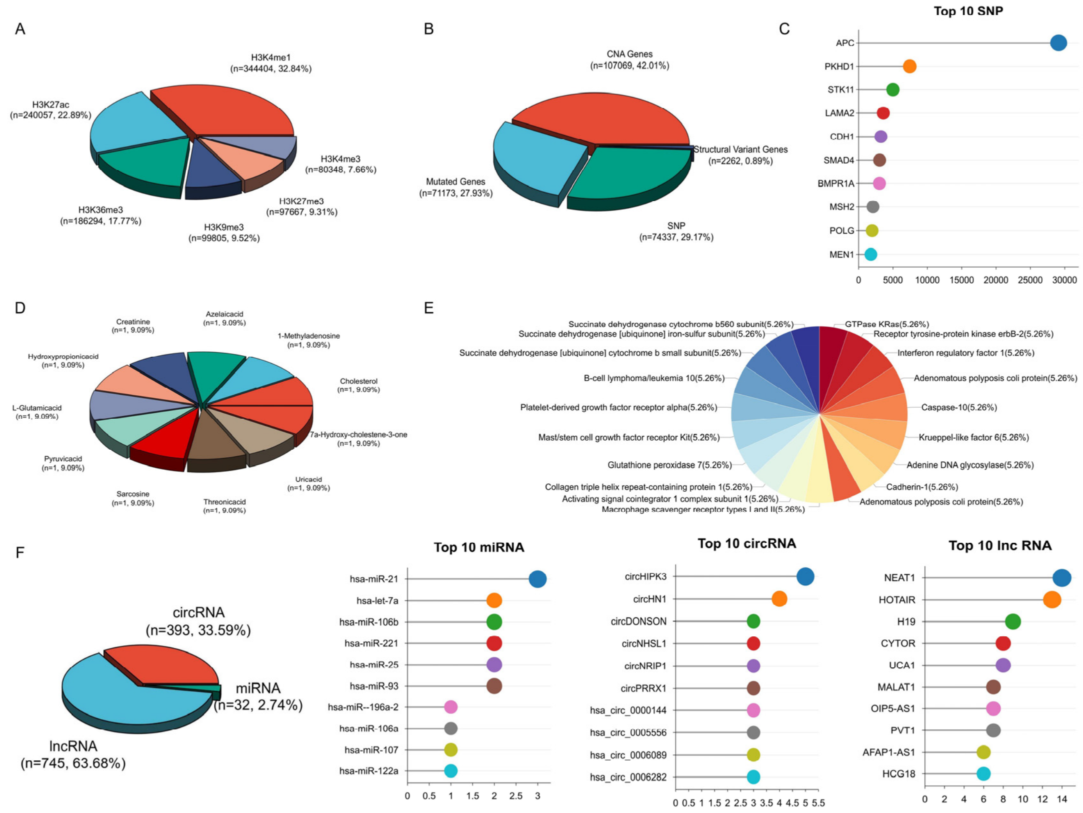

Supplementary Figure S2. Interactive visualization of multi-omics profiles associated with gastric diseases. (A) Pie chart summarizing six types of histone modifications. (B) Pie chart showing the distribution of genomic alterations. (C) Top 10 genes ranked by SNP count. The x-axis indicates the number of SNPs per gene, while the y-axis lists the top 10 genes; APC exhibits a markedly higher SNP count than the others, followed by genes in descending order. (D) Pie chart of 11 key metabolites associated with gastric cancer and atrophic gastritis. (E) Pie chart illustrating the functional features of proteins related to gastric diseases. (F) Pie chart and bar plots representing non-coding RNA profiles in the transcriptomic data, including the top 10 most abundant miRNAs, circRNAs, and lncRNAs.

Supplementary Table S1. Copy number alterations of CDH1 in different gastric-related diseases.

#	Gene	Disease	CNA	Cytoband	Freq	Is cancer gene	PMID
1	CDH1	Esophageal/Stomach Cancer	AMP	16q22.1	0.20%	Yes	33795256
2	CDH1	Esophageal/Stomach Cancer	HOMDEL	16q22.1	0.40%	Yes	33795256
3	CDH1	Esophagogastric Cancer	AMP	16q22.1	0.20%	Yes	37699004
4	CDH1	Esophagogastric Cancer	HOMDEL	16q22.1	0.40%	Yes	37699004
5	CDH1	Esophagogastric Cancer	AMP	16q22.1	0.40%	Yes	35377946
6	CDH1	Esophagogastric Cancer	HOMDEL	16q22.1	0.80%	Yes	35377946
7	CDH1	Esophagogastric Cancer	HOMDEL	16q22.1	1.60%	Yes	37406106
8	CDH1	Metastatic Esophagogastric Cancer	AMP	16q22.1	0.30%	Yes	29122777
9	CDH1	Metastatic Esophagogastric Cancer	HOMDEL	16q22.1	0.30%	Yes	29122777
10	CDH1	Gastric Cancer	HOMDEL	16q22.1	1.90%	Yes	29670109
11	CDH1	Gastric Cancer	AMP	16q22.1	0.90%	Yes	29670109
12	CDH1	Stomach Adenocarcinoma	HOMDEL	16q22.1	1.00%	Yes	25079317
13	CDH1	Stomach Adenocarcinoma	HOMDEL	16q22.1	1.10%	Yes	29625048<br>29596782<br>29622463<br>29617662<br>29625055<br>29625050<br>29617662<br>32214244<br>29625049<br>29850653
14	CDH1	Stomach Adenocarcinoma	AMP	16q22.1	0.20%	Yes	29625048<br>29596782<br>29622463<br>29617662<br>29625055<br>29625050<br>29617662<br>32214244<br>29625049<br>29850653

Supplementary Table S2. The chemical compound information included in the StomachDB database.

#	Gene Name	Disease Name	Species	Direct Evidence	Inference Network	Source	Reference Count
1	CMET	Gastric Fistula	Hunam	NA	Aspirin	CTD	1
2	PRH2	Gastric Fistula	Hunam	NA	Aspirin	CTD	1
3	SUGT1P3	Gastric Fistula	Hunam	NA	Aspirin	CTD	1
4	CA5BP1	Gastric Fistula	Hunam	NA	Aspirin	CTD	1
5	CLC	Gastric Fistula	Hunam	NA	Aspirin	CTD	1
6	PF4V1	Gastric Fistula	Hunam	NA	Aspirin	CTD	1
7	FAM27E3	Gastric Fistula	Hunam	NA	Aspirin	CTD	1
8	MIR1306	Gastric Fistula	Hunam	NA	Aspirin	CTD	1
9	GNLY	Gastric Fistula	Hunam	NA	Aspirin	CTD	1
10	TRAC	Gastric Fistula	Hunam	NA	Aspirin	CTD	1

Supplementary Table S3. The Chinese medicine information included in the StomachDB database.

#	Disease Name	Gene	Herbs Associated with This Disease	Website
1	Neoplasm Of The Stomach	ACD (HPO), AKT1 (HPO), APC (HPO), AXIN2 (HPO), BAP1 (HPO), BUB1B (HPO), CDK4 (HPO), CDKN2A (HPO), CDKN2B (HPO), CDKN2D (HPO), CTNNB1 (HPO), DCC (HPO), DLC1 (HPO), EP300 (HPO), FGFR3 (HPO), FLCN (HPO), KIT (HPO), MC1R (HPO), MGMT (HPO), MITF (HPO), MLH1 (HPO), MSH2 (HPO), MSH6 (HPO), NRAS (HPO), PDGFRA (HPO), PIK3CA (HPO), POT1 (HPO), SDHA (HPO), SDHB (HPO), SDHC (HPO), SRC (HPO), TERF2IP (HPO), TERT (HPO), TP53 (HPO)	YAO WANG CHA	http://www.tcmip.cn/ETCM/index.php/Home/Index/yc_details?ywname=YAO%20WANG%20CHA
2	Neoplasm Of The Stomach	ACD (HPO), AKT1 (HPO), APC (HPO), AXIN2 (HPO), BAP1 (HPO), BUB1B (HPO), CDK4 (HPO), CDKN2A (HPO), CDKN2B (HPO), CDKN2D (HPO), CTNNB1 (HPO), DCC (HPO), DLC1 (HPO), EP300 (HPO), FGFR3 (HPO), FLCN (HPO), KIT (HPO), MC1R (HPO), MGMT (HPO), MITF (HPO), MLH1 (HPO), MSH2 (HPO), MSH6 (HPO), NRAS (HPO), PDGFRA (HPO), PIK3CA (HPO), POT1 (HPO), SDHA (HPO), SDHB (HPO), SDHC (HPO), SRC (HPO), TERF2IP (HPO), TERT (HPO), TP53 (HPO)	MAI DONG	http://www.tcmip.cn/ETCM/index.php/Home/Index/yc_details?ywname=MAI%20DONG
3	Neoplasm Of The Stomach	ACD (HPO), AKT1 (HPO), APC (HPO), AXIN2 (HPO), BAP1 (HPO), BUB1B (HPO), CDK4 (HPO), CDKN2A (HPO), CDKN2B (HPO), CDKN2D (HPO), CTNNB1 (HPO), DCC (HPO), DLC1 (HPO), EP300 (HPO), FGFR3 (HPO), FLCN (HPO), KIT (HPO), MC1R (HPO), MGMT (HPO), MITF (HPO), MLH1 (HPO), MSH2 (HPO), MSH6 (HPO), NRAS (HPO), PDGFRA (HPO), PIK3CA (HPO), POT1 (HPO), SDHA (HPO), SDHB (HPO), SDHC (HPO), SRC (HPO), TERF2IP (HPO), TERT (HPO), TP53 (HPO)	DI YU	http://www.tcmip.cn/ETCM/index.php/Home/Index/yc_details?ywname=DI%20YU
4	Neoplasm Of The Stomach	ACD (HPO), AKT1 (HPO), APC (HPO), AXIN2 (HPO), BAP1 (HPO), BUB1B (HPO), CDK4 (HPO), CDKN2A (HPO), CDKN2B (HPO), CDKN2D (HPO), CTNNB1 (HPO), DCC (HPO), DLC1 (HPO), EP300 (HPO), FGFR3 (HPO), FLCN (HPO), KIT (HPO), MC1R (HPO), MGMT (HPO), MITF (HPO), MLH1 (HPO), MSH2 (HPO), MSH6 (HPO), NRAS (HPO), PDGFRA (HPO), PIK3CA (HPO), POT1 (HPO), SDHA (HPO), SDHB (HPO), SDHC (HPO), SRC (HPO), TERF2IP (HPO), TERT (HPO), TP53 (HPO)	ZHI SHI	http://www.tcmip.cn/ETCM/index.php/Home/Index/yc_details?ywname=ZHI%20SHI

Supplementary Table S4. The probiotic information contained in the StomachDB database.

#	Disease	Probiotics	Genus	Location	Function	PMID
1	Helicobacter pylori infection、gastroesophageal reflux disease	Lactobacillus johnsonii No. 1088	Lactobacillus	Gastric juice	Inhibits the growth of Helicobacter pylori, Escherichia coli O-157, Salmonella typhi and Clostridium difficile	25771812
2	Helicobacter pylori infection	Saccharomyces boulardii	Saccharomyces	Stomach	Improve diarrhea, upper abdominal discomfort and treatment tolerance, and reduce post-treatment dyspeptic symptoms	17669103
3	Helicobacter pylori infection	Lactobacillus acidophilus	Lactobacillus	Stomach	Improve eradication rates with standard Helicobacter pylori therapy	11121911
4	Helicobacter pylori infection	Bifidobacterium	Bifidobacterium	gastric mucosa	Altered gastric microbiota diversity, community structure, and composition	34448282
5	Helicobacter pylori infection	Lactobacillus	Lactobacillus	gastric juice	Altered gastric microbiota diversity, community structure, and composition	34448282
6	Gastrointestinal microbiota dysbiosis after Helicobacter pylori eradication	Bifidobacterium Tetragenous viable Bacteria Tablets	Bifidobacterium	Gastrointestinal tract	Regulate gastrointestinal microbiota, reduce gastrointestinal adverse events, and help microbiota recovery	36426355
7	Helicobacter pylori infection	Lactobacillus reuteri	Lactobacillus	Gastrointestinal tract	Improve eradication rates and reduce treatment-related adverse events	31554388

Supplementary Table S5. Comparison of features across StomachDB and representative existing databases.

Features	StomachDB	TCGA	GutUDB	gutMDisorder	GMrepo	AMDB	Twnbiome
Core Research Focus	Specialized in gastric diseases	Integration of multi-omics data and clinical information for cancer research	Integrated multi-omics analysis of gut diseases	Associations between gut microbiota, diseases, and interventions	Integration and standardization of human gut metagenomic data	Gut microbiomics research in animals	Baseline of gut microbiota in healthy populations in Taiwan
Covered Disease Types	Gastric cancer, gastritis, gastric ulcer, H. pylori-associated diseases; cancerous + non-cancerous	Over 33 types of cancers	56 gut-related diseases, including colorectal cancer, inflammatory bowel disease, irritable bowel syndrome, etc.	123 human diseases (e.g., digestive system diseases, diabetes, obesity) and 33 mouse diseases	Metabolic and inflammatory diseases	No specific disease types; focuses on host-microbe interactions	No direct disease association (serves as healthy control)
Integrated Omics Data Types	Genomics, transcriptomics, single-cell/spatial transcriptomics, proteomics, metabolomics, therapeutics	Genomics, transcriptomics, epigenomics, proteomics, metabolomics, clinical data	Epigenomics, genomics, transcriptomics, spatial omics, single-cell omics, proteomics, metabolomics, microbiomics	Microbiomics (16S rRNA sequencing, metagenomics)	Metagenomics, 16S rRNA sequencing	Microbiomics (16S rRNA sequencing, metagenomics)	Microbiomics (16S rRNA sequencing, metagenomics)
Single-Cell/Spatial Transcriptomics	Yes	Primarily bulk omics data; single-cell/spatial transcriptomics data are available only in	Yes	Not mentioned	Not mentioned	Not mentioned	Not mentioned

		derived resources					
Therapeutic-Related Data	Yes (31,413 chemicals; 1,950 TCM; 37 probiotics)	Comprehensive therapeutic data, including drug response, chemotherapy/radiotherapy regimens, and clinical treatment outcomes	Data on chemical drugs, traditional medicines, and probiotic preparations	77 human interventions and 151 mouse	Not explicitly stated, but some studies involve antibiotic treatment associations	Not explicitly stated	Not explicitly stated
Species Coverage	Human, Mouse	Human	Human, Mouse, Rat, Rhesus Monkey	Human, Mouse	Human	467 animal species	Human
Advantages	First dedicated, integrated multi-omics database for gastric diseases	The largest public cancer multi-omics database; strong integration of omics data and clinical information; supports cancer subtype classification and therapeutic target discovery	The first gut disease database covering 8 omics types; supports multi-omics visualization and personalized analysis	Integrates microbiota-disease-intervention ternary associations; supports data upload and cross-species comparison	The largest gut metagenomic database with 71,642 samples; supports cross-project comparison and disease biomarker identification	The first multi-species animal gut microbiota database; supports analysis of host diet and taxonomic levels	Provides reference for gut microbiota of local healthy populations; supports sample upload and online analysis
